# Impact of preoperative diastolic dysfunction on short-term outcomes following robotic-assisted minimally invasive esophagectomy (RAMIE)

**DOI:** 10.1007/s11701-025-02624-7

**Published:** 2025-08-01

**Authors:** Saeed Torabi, Philipp Omuro, Dolores T. Krauss, Sandra E. Stoll, Tobias Kammerer, Georg Dieplinger, Thomas Schmidt, Fabian Dusse, Andrea U. Steinbicker, Christiane J. Bruns, Lars M. Schiffmann, Hans F. Fuchs

**Affiliations:** 1 Department of Anesthesiology and Intensive Care Medicine, Medical Faculty of the University of Cologne, University Hospital of Cologne, Cologne, Germany; 2 Department of General, Visceral, Thoracic and Transplant Surgery, Medical Faculty of the University of Cologne, University Hospital of Cologne, Cologne, Germany

**Keywords:** Diastolic dysfunction, Robotic-assisted minimally invasive esophagectomy (RAMIE), Postoperative outcomes, Perioperative risk stratification, Preoperative transthoracic echocardiography

## Abstract

Diastolic dysfunction is a common echocardiographic finding in patients undergoing major surgery and has been associated with adverse perioperative outcomes, particularly in high-risk procedures. However, its prognostic relevance in robotic-assisted minimally invasive esophagectomy (RAMIE) remains unclear. This study investigates the impact of preoperative diastolic dysfunction on short-term postoperative outcomes and intensive care unit (ICU) course in patients undergoing RAMIE. A retrospective, monocentric cohort of 256 adult patients, who underwent robotic-assisted Ivor-Lewis esophagectomy for esophageal carcinoma at the Medical Faculty of the University of Cologne and University Hospital of Cologne (2019–2024), was screened. Of these, 181 cases with available preoperative transthoracic echocardiography (TTE) data were included in this study. Included patients were stratified based on the presence and grade of diastolic dysfunction in preoperative echocardiography. Postoperative outcomes including new-onset atrial fibrillation (POAF), pulmonary complications, anastomotic leakage, length of ICU stay, and mortality, were analyzed using χ2 and Kruskal–Wallis tests, with *p < 0.05 considered significant. 181 of 256 screened patients could be included in our study. Preoperative diastolic dysfunction was identified in 67 of 181 screened patients: 63 patients with grade I and 4 patients with grade II diastolic dysfunction. Patients with diastolic dysfunction were more likely to present with coronary artery disease (13 vs. 7, 19 vs. 6%; *p* = 0.01), diabetes mellitus (16 vs. 10, 24 vs. 9%; *p* = 0.01), and hypertension (37 vs. 43, 55 vs. 38%; *p* = 0.02) compared to those without. However, no differences were observed in postoperative outcomes, including postoperative atrial fibrillation (21 vs. 18%; *p* > 0.05), pulmonary complications (22% in both groups; *χ*^2^ = 0.045; *p* > 0.05), anastomotic leakage (16 vs. 18%; *χ*^2^ = 0.048, *p* > 0.05), ICU stay (median 2 days for both groups), or in-hospital mortality (4 vs. 2%; *p* > 0.05). The severity of complications, as classified by the Clavien–Dindo system, was also not associated with diastolic dysfunction (Pearson chi-square: *χ*^2^ = 1.094; *p* > 0.05). Mild diastolic dysfunction (predominantly grade I) was not associated with worse short-term outcomes in patients undergoing RAMIE. Despite a higher burden of cardiovascular comorbidities, ICU stay, postoperative complication rates, and mortality were comparable to patients with normal diastolic function. These findings suggest that mild diastolic dysfunction should not be considered a contraindication for RAMIE and highlight the need for refined risk stratification tools integrating echocardiographic and clinical parameter.

## Introduction

Cardiovascular comorbidities, particularly diastolic dysfunction have been identified as predictors of adverse postoperative outcomes in both cardiac and non-cardiac surgery [1–3]. Diastolic dysfunction is characterized by impaired left ventricular relaxation and elevated filling pressures, which may result in perioperative hemodynamic instability [4–6]. These abnormalities are associated with an increased risk of postoperative complications, including new-onset atrial fibrillation (AF), pulmonary edema, myocardial infarction, and anastomotic leakage, independent of left ventricular systolic function [2, 7].

These associations are especially relevant for high-risk procedures, such as Ivor-Lewis esophagectomy, which imposes considerable physiological and cardiovascular stress on patients [8]. Despite advances in surgical techniques, esophagectomy remains associated with substantial perioperative morbidity and mortality [8–11]. Among the most frequent cardiac complications is postoperative atrial fibrillation, occurring in up to 30% of cases [11–14]. Importantly, postoperative atrial fibrillation after Ivor-Lewis esophagectomy is strongly associated with increased postoperative complications, particularly pulmonary events, anastomotic leakage, and higher short-term mortality, underscoring its role as an early indicator of adverse outcomes [15].

Diastolic dysfunction is frequently underdiagnosed, as it often remains asymptomatic, yet its prevalence increases with age and is strongly linked to common cardiovascular risk factors such as hypertension, diabetes mellitus, and CAD [4, 16, 17]. Existing evidence suggests that diastolic dysfunction not only predisposes patients to perioperative cardiac complications [1, 2, 7, 18–20] but also contributes to increased long-term mortality [21–24]. With an aging surgical population and a growing burden of cardiovascular disease, the preoperative identification of diastolic dysfunction has become a critical step in modern perioperative risk assessment [25, 26].

Advanced echocardiography now plays an essential role in preoperative evaluation, enabling the detection of subtle diastolic impairments that may otherwise remain undetected in standard assessments [26]. This facilitates improved the risk stratification and allows for tailored perioperative management, which is crucial for optimizing outcomes in high-risk patients [5, 26, 27].

Furthermore, minimally invasive techniques such as robot-assisted esophagectomy have been shown to enhance postoperative recovery and reduce cardiopulmonary complications compared to open surgery, while maintaining equivalent oncological outcomes [28, 29].

This retrospective study aims to investigate the impact of diastolic dysfunction, primarily grade I, as identified through preoperative echocardiography, on postoperative outcomes following robot-assisted Ivor Lewis esophagectomy (RAMIE). Specifically, we analyze the incidence of new-onset AF, anastomotic leakage, ICU length of stay, and short-term mortality in patients with diastolic dysfunction compared to those with normal diastolic function. By exploring these associations, this study seeks to highlight the clinical relevance of comprehensive cardiac evaluation in the preoperative setting to improve perioperative management and surgical outcomes.

## Methods and statistics

### Ethical approval

This study was approved by the Ethics Committee of the University of Cologne, Germany (24-1319, 26.08.2024) with a waiver for the requirement of individual patient`s informed consent due to the retrospective character of the study and the analysis of anonymized data.

### Study design and setting

This retrospective single-center cohort study was conducted at the Medical Faculty of the University of Cologne and the University Hospital of Cologne, Germany, involving collaboration between the Departments of Anesthesiology and Intensive Care Medicine and the Department of General, Visceral, Thoracic, and Transplant Surgery. The study included patients from a surgical database, who underwent esophageal surgery between March 2019 and Juni 2024 and received postoperative Intensive Care at the University Hospital's Intensive Care Unit of the Department of Anesthesiology and Intensive Care Medicine.

### Patient’s selection

#### Inclusion criteria

All adult patients, who underwent elective robotic-assisted minimally invasive Ivor Lewis esophagectomy, characterized by either robotic or laparoscopic gastrolysis and robotic transthoracic esophagectomy, were included in this study.

#### Exclusion criteria

- Emergency esophagectomy for trauma or Boerhaave syndrome
- McKeown esophagectomy with cervical anastomosis
- benign indications such as achalasia
- open esophagectomies
- Patients, who initially underwent robotic-assisted esophagectomy, but required intraoperative conversion to open surgery, were also excluded from the study

### Surgical technique

We have previously published a detailed treatment pathway of patients with esophageal cancer at our clinic [30]. As most patients present with a locally advanced tumor, following a multimodal treatment pathway, surgery is performed 4–6 weeks after completion of neoadjuvant treatment and restaging diagnostics. An Ivor Lewis esophagectomy with reconstruction using a gastric conduit and high intrathoracic anastomosis depicts the current standard and is performed in a totally minimally invasive fashion using the DaVinci Xi robotic surgical system (Intuitive Surgical Inc, Sunnyvale, CA. USA). The thoracic part is always performed robotically, since the advantage of the precise resection using the robotic device becomes evident during the thoracic lymphadenectomy. The abdominal part can be performed robotically as well or in a laparoscopic way. We have previously published our standardized surgical technique in detail in the following manuscript [30].

Thus selection of patients for the current trial ensured a homogeneous patient cohort undergoing a standardized surgical approach (RAMIE).

Moreover, all patients included in this study received a preoperative cardiac assessment in form of a preprocedural echocardiography and all patients included in this study were admitted to the Intensive Care Unit postoperatively.

At our institution, preoperative transthoracic echocardiography (TTE) is routinely performed in all patients aged ≥ 60 years or in those with known or suspected cardiovascular comorbidities, such as hypertension, coronary artery disease, or diabetes mellitus, primarily in patients classified as ASA III. Consequently, patients who do not undergo TTE likely represent a lower-risk cohort.

All patients undergoing RAMIE are evaluated by a multidisciplinary tumor board and receive standardized preoperative preparation, including ECG, pulmonary function testing, cardiology consultation, and transthoracic echocardiography (TTE). The indication for TTE is based on the Charlson Comorbidity Index (CCI), patient age, the presence of cardiovascular comorbidities, and at the discretion of the preoperative anesthesia consultation. This preoperative assessment is conducted in accordance with the ESC guidelines for non-cardiac surgery [31].

### Data collection and statistical analyses

Adult patient who received RAMIE due to esophageal cancer between 2019 and 2024 at the University hospital of Cologne were screened for study inclusion using Data warehouse eisTIK (KMS GmbH, Unterhaching, Germany).

Perioperative data were retrospectively collected from a prospectively maintained surgical database. Electronic data sources included the in-hospital’s electronic and paper-based documentation from patients’ charts, patients’ files, and anesthesia records.

Anonymized data were transferred into Microsoft Excel. For statistical analysis IBM^®^ SPSS^®^ Statistics 29.0.2.0 (New York, USA 2023) was used. Depending on the type of data *χ*^2^- or Kruskal–Wallis-tests were used. A *p < 0.05 was considered as significant. Data are displayed as mean [25.–75. percentile] or relative frequency % (*n* = absolute frequency).

Data extraction included:

- Demographic data: Age, sex, and relevant comorbidities
- Preoperative diagnostic: Transthoracic echocardiography (TTE)
- Surgical variables: Surgical approach (robotic assisted Ivor-Lewis), duration of surgery, and duration of one-Lung ventilation and the amount of intraoperative blood loss
- Postoperative outcomes: Incidence of hemodynamically relevant postoperative atrial fibrillation, incidence of pulmonary complications and anastomotic leakage, ICU length of stay, and in-hospital mortality.

Definition of diastolic dysfunction [4, 26]:

The diagnosis of diastolic dysfunction relies on several key echocardiographic parameters that assess myocardial relaxation, filling pressures, and ventricular compliance. Central to this evaluation are mitral inflow parameters**,** including the E/A ratio and deceleration time (DT), which reflect the pattern and duration of diastolic filling. Tissue Doppler Imaging (TDI) provides critical insights into myocardial relaxation through the *e*′ velocity and the *E*/*e*′ ratio, which is an important surrogate marker for left ventricular filling pressures. The left atrial (LA) volume index serves as an indicator of chronically elevated filling pressures, while pulmonary vein flow patterns (systolic and diastolic components, S/D ratio) further elucidate diastolic function. Tricuspid regurgitation (TR) velocity aids in estimating pulmonary artery pressures, which are often elevated in advanced diastolic dysfunction. Additionally, the isovolumetric relaxation time (IVRT) provides a measure of the time required for ventricular relaxation, and global longitudinal strain (GLS) detects early myocardial dysfunction, even in the presence of normal ejection fraction.

Assessment and diagnosis of diastolic dysfunction [4]:

The assessment and diagnosis of left ventricular diastolic dysfunction requires a structured approach that incorporates various echocardiographic parameters. Based on current guidelines from the British Society of Echocardiography (BSE), the severity of diastolic dysfunction can be classified in Grad I: Impaired Relaxation, Grade II: Pseudonormalization, Grade III: Reversible Restrictive Filling and Grade IV: Fixed Restrictive Filling [4].

All echocardiographic assessments were performed by board-certified cardiologists at our university hospital following BSE guidelines. All echocardiographic findings were subsequently validated by intensivists within our working group.

## Results

A total of 256 patients, who underwent robotic-assisted esophagectomy for esophageal cancer at the Medical Faculty of the University of Cologne and the University Hospital of Cologne between March 2019 and June 2024 were screened. 75 cases without available preoperative transthoracic echocardiography (TTE) were excluded. A total of 181 cases were included in the final analysis, of which preoperative diastolic dysfunction was identified in 67 patients (63 with grade I dysfunction and only 4 with grade II dysfunction). The remaining 114 patients showed no evidence of diastolic dysfunction. All 4 patients with grade II diastolic dysfunction were managed by the cardiology team prior to surgery. Preoperative optimization included intensified antihypertensive therapy and in one case initiation of diuretics. We also clarify that no patient had symptomatic heart failure or elevated NT-proBNP.

Preoperative transthoracic echocardiography (TTE) was not associated with a reduced risk of major postoperative complications (Clavien–Dindo classification). However, patients who underwent preoperative TTE showed a significantly higher incidence of postoperative atrial fibrillation (POAF) (*χ*^2^ test, *p* = 0.019). This may reflect preselection of higher-risk patients for preoperative TTE. No difference was observed in postoperative length of stay.

### Patients’ preoperative characteristics

The study cohort had a median age of 63 years [57–70]. Age, height, or gender distribution between patients with and without diastolic dysfunction were not different (Table 2).

Patients diagnosed with diastolic dysfunction, however, demonstrated notable differences in certain characteristics (Table 2). These individuals had a higher body weight (85 [72–91] vs. 79 [70–87] kg; *p* = 0.03) and body mass index (BMI) (26.1 [23.6–28.7] vs. 24.8 [22.6–26.8]; *p* = 0.02) compared to those without diastolic dysfunction.

In terms of comorbidities, diastolic dysfunction was associated with a higher prevalence of coronary artery disease (19 vs. 6%; *p* = 0.01), diabetes mellitus (24 vs. 9%; *p* = 0.01), and arterial hypertension (55 vs. 38%; *p* = 0.02). Other comorbid conditions, including atrial fibrillation, hepatic disease, chronic kidney disease, pulmonary disease, and neurologic disorders, showed no differences between the two groups (Table 1.) Additionally, preoperative pulmonary function metrics, such as FEV1, FVC, and the Tiffeneau Index, did not show differences between patients with and without diastolic dysfunction (FEV1 3.4 [2.93–3.66] vs. 3.37 [2.96–3.71] L; H = 0.120; FVC 4.20 [3.83–4.79] vs. 4.47 [3.91–4.88] L; H = 0.201; Tiffenau Index 76.86 [75.70–78.05] vs. 77.22 [75.72–78.23] %; H = 0.753).

**Table 1:** Preoperative characteristics of all patients as well as patients with and without preoperative diastolic dysfunction

	All (*n* = 181) Mean (interquartile range) % (*n*)	Non diastolic dysfunction (*n* = 114) Mean (interquartile range) % (*n*)	Diastolic dysfunction (*n* = 67) Mean (interquartile range) % (*n*)	*H* or *χ*^2^	*p*-value
Age (years) (min–max)	63 [57–70]	63 [56–68]	65 [58–70]	H 1.996	n.s.
BMI (kg/m^2^) Mean (25.–75. percentile)	25.3 [22.9–27.8]	24.8 [22.6–26.8]	26.1 [23.6–28.7]	H 5.340	0.02
Gender male/female	79%/21% (143/38)	77%/23% (88/26)	82%/18% (55/12)	*χ*^2^ 0.610	n.s.
ASA-Score	I 19% (35) II 54% (97) III 26% (47)	I 21% (24) II 55% (63) III 22% (25)	I 16% (11) II 45% (30) III 33% (22)	*χ*^2^ 6.012	n.s.
Coronary artery diseases	11% (20)	6% (7)	19% (13)	*χ*^2^ 7.78	0.01*
Atrial fibrillation	10% (18)	7% (8)	15% (10)	*χ*^2^ 2.95	n.s.
Diabetes mellitus	14% (26)	9% (10)	24% (16)	*χ*^2^ 7.83	0.01*
Arterial hypertension	44% (80)	38% (43)	55% (37)	*χ*^2^ 5.243	0.02*
Hepatic disease	7% (12)	7% (8)	6% (4)	*χ*^2^ 0.075	n.s.
Neurologic disease	7% (13)	7% (8)	7% (5)	*χ*^2^ 0.013	n.s.
COPD	14% (26)	11% (13)	19% (13)	*χ*^2^ 2.195	n.s.

Mean [25.–75. percentile] or relative frequency % (*n* = absolute frequency)

*BMI* body mass index, *COPD* chronic obstructive pulmonary disease, *ASA* American Society of Anesthesiology, *H* = Kruskal–Wallis *H*, *χ*^2^ = Chi^2^-test,* n.s.* non-significant

*p < 0.05 = significant

Intraoperative surgical parameters were similar between the two groups. The median operative duration (342 [307–396] vs. 355 [317–410] minutes; *H* = 2.840; *p* > 0.05), one-lung ventilation time (170 [140–201] vs. 180 [150–210] minutes; *H* = 3.215; *p* > 0.05), and perioperative blood loss (200 [100–300] ml in both groups; *p* > 0.05) were comparable. Of note, we did not expect differences in surgical duration due to diastolic dysfunction of the patients.

### Postoperative outcomes

The analysis of postoperative outcomes revealed no differences between patients with and without preoperative diastolic dysfunction regarding key postoperative outcomes, including the occurrence of POAF, the severity of complications (Clavien–Dindo classification), or specific complications such as pulmonary events or anastomotic leakage (Table 2).

**Table 2:** Outcome parameters of all patients as well as patients without and with diastolic dysfunction

	All (*n* = 181) Mean (interquartile range) % (*n*)	Normal diastolic function (*n* = 114) Mean (interquartile range) % (*n*)	Diastolic dysfunction (*n* = 67) Mean (interquartile range) % (*n*)	*H* or *χ*^2^	*p*-value
Length of ICU stay (days)	2 [2–4]	2 [2, 3]	2 [2–4]	*H* 0.335	n.s.
Length of hospital stay	14 [12–21]	14 [12–20]	14 [13–21]	*H* 0.063	n.s.
ICU readmission rate	19% (34)	20% (23)	16% (11)	*χ*^2^ 0.425	n.s.
In-hospital mortality	3% (5)	2% (2)	4% (3)	*χ*^2^ 1.142	n.s.
POAF	21% (38)	18% (21)	25% (17)	*χ*^2^ 1.164	n.s.

Mean [25.–75. percentile] or relative frequency % (*n* = absolute frequency)

*H* Kruskal–Wallis *H*, *χ*^2^ Chi^2^-test, *n.s.* non-significant

*p* < 0.05 = significant

The median length of ICU stay was 2 [2–4] days in both groups and the length of in-hospital stay was also comparable with 14 [12–21] days in both groups. Similarly, ICU readmission rates (16 vs. 20%) and mortality (4 vs. 2%) showed no differences among both groups. Diastolic dysfunction, predominantly grade 1 in this patient cohort, did not influence the occurrence of POAF, with rates of 25% in patients with diastolic dysfunction compared to 18% in those without. Of the 38 patients who developed POAF, 7 (18%) also experienced anastomotic leakage. This overlap was not statistically significant (*p* = 0.42).

The rates of postoperative pulmonary complications (22% in both groups), rate of surgical revisions (13 vs. 6%), and anastomotic leakage or conduit ischemia (16 vs. 18%) were also within a similar range in both groups. Furthermore, there was no association between diastolic dysfunction and the severity of postoperative complications classified as Clavien–Dindo major vs. minor. These findings indicate that preoperative mild diastolic dysfunction of grade 1 does not independently affect postoperative outcomes in patients undergoing robotic-assisted esophagectomy.

To conclude, the data of our study showed that mild diastolic dysfunction was more prevalent in patients with coronary artery disease, atrial fibrillation, diabetes, and hypertension. Nevertheless, these comorbidities did not translate into significantly different surgical or postoperative outcomes.

## Discussion

This current study aimed to explore the impact of a preoperative diastolic dysfunction, as assessed by TTE, on postoperative outcomes in patients undergoing RAMIE. The findings revealed that preoperative diastolic dysfunction grade 1 was more prevalent in patients with coronary artery disease, atrial fibrillation, diabetes and hypertension, but was not associated with worse specific postoperative outcomes, including new-onset atrial fibrillation (POAF), lengths of ICU stay, or the severity of complications according to the Clavien–Dindo classification.

Advanced echocardiographic techniques, including Doppler flow and strain imaging, have enabled the assessment of diastolic dysfunction [4, 32, 33]. These tools allow the identification of subtle cardiac impairments, aiding in the stratification of the perioperative risk [33]. However, Nagueh et al. mentioned that while echocardiography plays an important role in perioperative risk assessment, diastolic dysfunction alone may not independently contribute to worsen short-term postoperative outcomes [26]. This was also reflected in our study. While diastolic dysfunction was associated with a higher prevalence of comorbidities, such as coronary artery disease, atrial fibrillation, diabetes mellitus, and hypertension, these conditions did not translate into increased rates of postoperative complications such as POAF or short-term mortality. However, the analyzed outcomes are only in the context of short-term outcomes. It is conceivable that long term outcomes will be impaired in patients with more comorbidities and diastolic dysfunction, for example 1-years survival. Willingham et al. [2] reached similar conclusions that diastolic dysfunction does not appear to be associated with increased in-hospital mortality, acute kidney injury, or hospital length of stay in a cohort of non-cardiac surgical patients [2]. This contrasts with prior studies suggesting a strong link between diastolic dysfunction of any grade and adverse surgical outcomes [34], particularly in high-risk procedures [18, 35]. Other studies were even able to show that the intraoperative detection of a diastolic dysfunction can potentially exacerbate the risk of hemodynamic instability postoperatively [36, 37], leading to complications such as atrial fibrillation and reduced tissue perfusion [37]. However, these findings were not reflected in our study cohort, most probably due to the mild diastolic dysfunction grade I and II in this patient cohort.

POAF is a well-documented complication following esophagectomy, with reported incidences of up to 30% [38, 39]. Despite the increased cardiovascular burden in patients with diastolic dysfunction [40–42], in our patient cohort the incidence of POAF was similar to patients without diastolic dysfunction. Of the 38 patients who developed POAF, 7 (18%) also experienced anastomotic leakage. This overlap was not statistically significant (*p* = 0.42), but consistent with prior literature suggesting POAF may be an early marker of further postoperative complications, like anastomotic leakage [15].

The procedure esophagectomy per se may predispose to POAF, as intrathoracic surgery takes place and pressure, inflammation, and single-lung ventilation may stress the heart to develop POAF. Pre-existing cardiac conditions, such as hypertension are often considered risk factors for the development of POAF [43], particularly in older patients. Given the established recognition of new-onset POAF as a significant predictor of complications such as pulmonary issues [44, 45] and anastomotic leakage [46, 47], it is highly recommended to perform a comprehensive preoperative risk assessment. This should include an evaluation of cardiac functions, with particular attention to diastolic function.

Left atrial diameter (LAD) and *E*/*e*′ ≥ 8.4 have been identified as independent risk factors for the development of POAF [48]. An elevated *E*/*e*′ indicates increased left atrial pressure, which is commonly associated with grade II or higher diastolic dysfunction. In our cohort, POAF was observed in 38 patients. The distribution of diastolic dysfunction grade 1 (*n* = 63) and grade 2 (*n* = 4) may explain the lack of an association between diastolic dysfunction and POAF in our analysis. Among the four patients with grade II diastolic dysfunction, all developed postoperative atrial fibrillation (POAF). While the sample size was too small for statistical testing, this observation may indicate a stronger association between advanced DD and POAF risk. As highlighted in a recent meta-analysis there is a significant association between the severity of preoperative diastolic dysfunction and the risk of postoperative complications [1].

Anastomotic leakage remains a critical complication following esophagectomy, often associated with increased morbidity and prolonged hospitalization. An analysis conducted by the Society of Thoracic Surgeons revealed that heart failure, coronary artery disease, and hypertension are predictors of anastomotic leakage following esophagectomy [47]. Due to the potentially life-threatening consequences of anastomotic leakage, risk assessment tools such as the NUn-Score [49] have been developed. These tools serve as predictive instruments to facilitate early diagnosis. The NUn-Score has been extensively validated [50–54]and primarily focuses on inflammatory processes and postoperative CRP elevation. Additionally, Goense et al. demonstrated that other risk factors, such as cardiac comorbidities, emphasize the interplay between impaired cardiac output, tissue perfusion, and anastomotic healing [55].

The median ICU length of stay, and in-hospital mortality rates were comparable between patients with and without diastolic dysfunction in our study. In contrast, Luitel et al. demonstrated that critically ill patients in the ICU with left ventricular diastolic dysfunction (LVDD) not only present a higher incidence of weaning failure but also experience increased short-term mortality [56]. Bursi et al. [40] concluded that symptomatic diastolic dysfunction carries a high mortality rate [40]. In contrast, Vignon et al. demonstrated that while LVDD was highly prevalent among patients with septic shock, it was not associated with an increase in mortality [21]. Notably, one-third of survivors after septic shock showed improvement in diastolic function over time [21]. The contrasting findings of these studies from Luitel [56], Bursi [40] and Vignon et al. [21], regarding the effect of diastolic dysfunction on mortality are likely attributable to differences in patient cohorts and study designs. In our cohort, 34% of patients with diastolic dysfunction were classified as grade I and underwent elective surgery for cancer and underwent pre-operative cardiologic assessment and cardiac improvement when needed. The study population of Luitel et al. [56] was older and admitted to the ICU due to different etiologies and were already mechanically ventilated for more than 48 h. The study by Vignon et al. [21] primarily focused on patients in septic shock, analyzing the impact of diastolic dysfunction on outcomes in this critically ill population [21].

The meta-analysis by Kaw et al. also reported higher mortality rates, but specifically among patients with grade III diastolic dysfunction [1]. The analysis of twelve studies included in this meta-analysis demonstrated that the severity of preoperative diastolic dysfunction is significantly associated with an increased risk of postoperative mortality following cardiovascular surgery [1]. In contrast, Willingham et al. demonstrated in their study of more than 12,871 non-cardiac surgical patients that diastolic dysfunction, regardless of its severity, was not associated with in-hospital mortality [2].

Our findings contrast with those of Kaw et al., who reported significantly increased mortality and complication rates in patients with diastolic dysfunction undergoing cardiac surgery [1]. This discrepancy is likely attributable to differences in surgical invasiveness, patient population, and diastolic dysfunction severity. While Kaw et al. included a high proportion of patients with moderate to severe (grade II–IV) DD undergoing high-risk cardiac procedures, our cohort was primarily composed of patients with mild (grade I) dysfunction undergoing standardized, minimally invasive oncologic surgery (RAMIE). Thus, the prognostic relevance of DD appears to be procedure-specific and severity-dependent.

The use of novel biomarkers [57], such as NT-proBNP [58], in conjunction with echocardiographic findings may further enhance the risk stratification and have already been implemented in national guidelines [59].

The small sample size and monocentric study design of retrospective nature may introduce selection bias limiting causal inference. One important limitation of our study is the lack of statistical power to detect small differences in the incidence of postoperative atrial fibrillation (POAF) between patients with and without diastolic dysfunction. Although the observed POAF rates were numerically higher in the diastolic dysfunction group (25 vs. 18%), a post-hoc power analysis revealed that a total sample size of approximately 689 patients would be required to detect this difference with 80% power at a significance level of 0.05. The absence of statistical significance in our study may, therefore, reflect insufficient sample size rather than a true lack of association. Future larger multi-center studies are necessary to further clarify this relationship. The exclusion of 75 cases without TTE data reduced the overall sample size.

In addition, the patients presented predominantly with diastolic dysfunction grade 1, only a few with grade 2, but none with grade 3 and 4. Therefore, our conclusions can only be associated to mild diastolic dysfunction. In contrast, we focus on an extremely homogeneous cohort undergoing a standardized surgical procedure with low complication rates overall.

## Conclusion

Our study demonstrated a higher prevalence of diastolic dysfunction of grade 1 in patients with coronary artery disease, diabetes mellitus, and hypertension. However, there was no association between preoperative diastolic dysfunction grades I and II and postoperative outcomes in patients undergoing robotic-assisted Ivor-Lewis esophagectomy. This may be attributed to the predominance of mild diastolic dysfunction (grade I) in our cohort and the standardized minimally invasive surgical approach used. Therefore, diastolic dysfunction should not be considered an exclusion criterion in the preoperative assessment for robotic assisted minimally invasive esophagectomy (RAMIE). Due to the predominance of mild (grade I) diastolic dysfunction and the absence of grades III and IV in our cohort, conclusions are limited to early-stage DD.

Our findings support the feasibility and safety of RAMIE in patients with mild (predominantly grade I) diastolic dysfunction. These results suggest that mild DD should not be viewed as a contraindication to minimally invasive esophagectomy but rather integrated into a structured preoperative assessment framework. This study provides reassurance for clinicians that patients with mild DD may proceed with RAMIE after appropriate cardiologic evaluation and optimization, without an expected increase in short-term complications.

Future research involving larger multi-center studies with long-term outcomes with a broader range of diastolic dysfunction grades and ages should further investigate its impact on clinical outcomes, explore potential interventions, and refine risk stratification strategies.

## Data Availability

The datasets used and analyzed during the current study are available from the corresponding author upon reasonable request.
